# Lie to me to lay with me: Females deceive males via terminal investment

**DOI:** 10.1371/journal.pone.0301942

**Published:** 2024-07-08

**Authors:** Laura Mendoza-Díaz de León, Sagrario Cordero-Molina, Indikris Krams, Jorge Contreras-Garduño

**Affiliations:** 1 Escuela Nacional de Estudios Superiores, Unidad Morelia, Universidad Nacional Autónoma de México, Mexico City, Mexico; 2 Latvian Biomedical Research and Study Centre, Riga, Latvia; 3 Institute of Ecology and Earth Sciences, University of Tartu, Tartu, Estonia; 4 Department of Zoology and Animal Ecology, Faculty of Biology, University of Latvia, Rīga, Latvia; 5 Institute for Evolution and Biodiversity, University of Münster, Münster, Germany; National Institute of Agricultural Research - INRA, MOROCCO

## Abstract

Historically, males have frequently been portrayed as the manipulative and deceptive gender, while females are often seen as adopting a coy and passive role. In this context, it is proposed that males use a terminal investment strategy, misleading females about their true poor condition, while females passively opt to mate with these deceptive males. However, we hypothesize that females in suboptimal condition may also engage in a terminal investment strategy by mimicking or enhancing their attractiveness to match that of females in better conditions. We studied this hypothesis in *Tenebrio molitor*, by subjecting females to three varying doses of lipopolysaccharides of *Escherichia coli* (LPS; 0.25, 0.5, or 1 mg ml^-1^), or three doses of the pro-oxidant Paraquat (PQ; 20, 40 or 80 mM), and subsequently assessing their survival and attractiveness to males. The LPS treatments and 20 mM of PQ had no significant effect on the survival or attractiveness of the females. However, females treated with 40 or 80 mM PQ survived fewer days compared to the control group. Those injected with 40 mM were more attractive than their control counterparts, while those treated with 80 mM were less attractive. Since the identical doses of LPS, which induce terminal investment in males, had no effect on females, we suggest sexual dimorphism in terminal investment. Furthermore, similar to males, if the stressor reaches a sufficiently high level, the signal becomes honest. These findings highlight how the quantity of stressors influences support for the terminal investment strategy in both males and females. Notably, this study challenges prevailing notions regarding gender roles in sexual selection, indicating that females, not just males, conceal their poor condition to attract mating partners.

## 1. Introduction

Since Darwin [1], sexual selection theory has largely emphasized reinforcing traditional sex roles while overlooking the active role of females in mating outcomes [2]. Given that optimal reproduction for males and females doesn’t always align, males may evolve manipulative strategies to enhance mating success at the expense of female fitness [3–5]. However, recent findings suggest that females are not as passive as previously thought [6, 7]. Instead, they can actively employ reproductive strategies, including engaging in multiple mating [8], and males may choose mates based on female attractiveness across various taxa [9]. Engaging in multiple mating provides females with advantages such as increased genetic diversity in offspring [10, 11], reduced risk of genetic incompatibility [12], and competition among sperm from diverse males, resulting in selection for high-quality sperm [13]. Additionally, females may obtain material benefits from males, such as access to resources, protection, parental care [6], or even beneficial molecules in the ejaculate [11, 14]. Nonetheless, whether females might adopt an offensive role and deceive males about their condition to mate with them remains largely unexplored [10, 11].

Most research on sexual selection assumes males are the courting sex and females are the choosing sex, leading to less emphasis on exploring female sexual traits as condition-dependent signals [15]. However, this approach overlooks that males also use female ornaments for mate choice [9, 16]. For instance, in the pink bollworm, *Pectinophora gossypiella*, the pheromone blend produced by females varies depending on their condition, including factors such as size, nutritional state, and age [17]. Male moths prefer females that produce more pheromones, which also correlates with higher egg production compared to females with lower pheromone levels [17], indicating that females provide honest signals about their condition [6]. However, from the perspective of terminal investment, organisms may allocate resources towards immediate reproduction at the expense of future reproduction when survival is uncertain [18]. For example, females facing imminent death invest in traits such as egg number, litter size, oviposition rate, hatching time, parental care, or food provisioning to their offspring (Table 1). Nonetheless, the hypothesis regarding whether females in poor condition, employing a terminal investment strategy, become more attractive to males when facing mortality remains unexplored. We suggest that females, not only males, may hide their condition and manipulate the reproductive interest of the opposite sex.

**Table 1:** Shows that terminal investment used by females to deceive males is a novel area of research, as no studies have ever focused on females cheating to attract males. We selected studies from Duffield (2017) and literature published between 2018 and September 2022 regarding terminal investment.

Phylum	Species	Sex	Challenge	Response	Effect	Reference
Mollusca	*Biomphaleria glabrata*	F	Immune	Size	Mixed (dependent on time)	Minchella & Loverde, 1981 [19]
Chordata	*Larus califonicus*	F	Age	F2 success; behaviour	Young < middle aged < old	Pugesek *et al*. 1981 [20]
Chordata	*Ovis canadensis*	F	Age	Behaviour	Increase with age	Festa-Bianchet, 1988 [21]
Chordata	*Ficedula albicollis*	F	Age	Behaviour	Young < old	Pärt *et al*. 1992 [22]
Arthropoda	*Acheta domesticus*	F	Immune	Size	Treatment > control	Adamo, 1999 [23]
Chordata	*Ficedula hypoleuca*	F	Immune	Time	Treatment > control; mixed	Sanz *et al*. 2001 [24]
Chordata	*Oreamnos americanus*	F	Age	Size	Increase with age	Côté & Festa-Bianchet, 2001 [25]
Chordata	*Alces alces*	F	Age	Size	Increase with age	Ericsson *et al*. 2001 [26]
Chordata	*Passer domesticus*	F	Immune	Time; F2 success	Treatment > control	Bonneaud *et al*. 2004 [27]
Arthropoda	*Daphnia magna*	F/M	Immune	Size	Mixed	Chadwick & Little, 2005 [28]
Arthropoda	*Gryllus texensis*	F	Immune	Size	Treatment > control	Shoemaker *et al*. 2006 [29]
Chordata	*Sorrateria mollissima*	F	Immune	Behaviour	Treatment > control	Hanssen, 2006 [30]
Arthropoda	*Gryllus texensis*	F	Age	Size	Increase with age	Shoemaker *et al*. 2006 [29]
Mollusca	*Biomphaleria glabrata*	F	Immune	Size; F2 success	Mixed (dependent on dose)	Blair & Webster, 2007 [31]
Chordata	*Chrysemys picta*	F	Age	Size; F2 success	Young < old	Paitz *et al*. 2007 [32]
Chordata	*Tamiasciurus hudsonicus*	F	Age	Time; F2 success	Increase with age	Descamps *et al*. 2007 [33]
Chordata	*Delicha urbica*	F/M	Immune	Time; Size; F2 success	Treatment > control	Marzal *et al*. 2008 [34]
Chordata	*Peromyscus mentculatus*	F	Immune	Size; F2 success	Treatment > control	Schwanz e*t al*. 2008 [35]
Arthropoda	*Acyrthosiphon pisum*	F	Immune	Size	Treatment > control; mixed	Altincicek *et al*. 2008 [36]
Chordata	*Peromyscus mentculatus*	F	Immune	Size	Treatment > control	Schwanz, 2008 [37]
Chordata	*Kobus megaceros*	F	Age	Size; sex ratio	Increase with age	Bercovitch *et al*. 2009 [38]
Arthropoda	*Nicrophorus orbicollis*	F	Age	Size; behaviour	Mixed	Creighton *et al*. 2009 [39]
Chordata	*Cervus elaphus*	F	Age	Size	Increase with age	Clutton-Brock *et al*. 1982 [40]
Arthropoda	*Nauphoeta cinera*	F	Diet	Size	Mixed	Barrett *et al*. 2009 [41]
Arthropoda	*Nicrophorus vespilloides*	F	Immune	F2 success	Treatment > control	Cotter *et al*. 2011 [42]
Chordata	*Macaca mulatta*	F	Age	Behaviour	Increase with age	Hoffman, 2010 [43]
Chordata	*Troglodytes aedon*	F	Immune	Size; sex ratio	Treatment > control	Bowers *et al*. 2012 [44]
Arthropoda	*Cardiocondyla obscurior*	F	Age	Size	Increase with age	Heinze & Schrempf, 2012 [45]
Chordata	*Troglodytes aedon*	F	Immune	Size; F2 success	Treatment > control	Bowers *et al*. 2015 [46]
Arthropoda	*Heliothis verescens*	F	Immune	Size (mixed); behaviour	Mixed	Staudacher *et al*. 2015 [47]
Chordata	*Litoria verreauxii alpina*	F/M	Immune	Size	Treatment > control	Brannelly *et al*. 2016 [48]
Arthropoda	*Cardiocondyla obscurior*	F	Immune	Size	Treatment > control	Giehr *et al*. 2017 [49]
Nematoda	*Heligmosomoides vespilloides*	F	Immune	Size	Treatment > control	Guivier *et al*. 2017 [50]
Arthropoda	*Gryllus texensis*	F	Predator simulation	Size	Treatment > control	Adamo & McKee, 2017 [51]
Chordata	*Taeniopygia guttata*	F	Immune	Size	Treatment > control	Sköld-Chiriac *et al*. 2019 [52]
Arthropoda	*Drosophila melanogaster*	F	Diet + immune	Size	Treatment > control	Hudson *et al*. 2020 [53]
Phylum	Species	Sex	Challenge	Response	Effect	Reference
Mollusca	*Biomphaleria glabrata*	F	Immune	Size	Mixed (dependent on time)	Minchella & Loverde, 1981 [19]

The table includes the sex studied, species, challenge, response, and effect. The response was classified as “size,” encompassing modifications in egg number, litter or egg size/mass, oviposition rate, and changes in oviduct or ovary size. For “time”, we included changes in the time to the first reproductive event or time to hatching. “Sex ratio” refers to the ratio of females to males or the resource allocation to one sex over other (larger mass/size for one sex). For “F2 (second generation) success”, we included results showing changes in hatching success, survival, or size to adulthood. The “behavioural” category encompasses alterations in parental care, such as variations in lactation time and shifts in oviposition sites. We excluded 18 studies (from 1988–2018) that looked for terminal investment in females but did not find evidence for it. So, only 56.09% of studies support the terminal investment by females.

To test the hypothesis, we used the mealworm beetle *Tenebrio molitor*. This species is promiscuous, exhibiting no morphological differences between sexes in colour and shape, and both sexes engage in mate choice [54, 55]. Mate choice and sexual communication occur through chemical signals, as males and females produce odours simultaneously to facilitate mating interactions [56]. On the one hand, males that produce a higher quantity of the pheromone (Z)-3-dodecenyl acetate (Z3-12: Ac) [57] are preferred by females compared to those producing it in lower levels [58]. This could be explained because pheromones reveal the resistance to infections and immunocompetence of their bearers [58–63], resistance to oxidative stress [55], and their nutritional status [61]. On the other hand, females produce the pheromone 4-methyl-1-nonanol [64], which reveals to potential mates their reproductive status [65] and age [66].

It has been widely demonstrated that males use the terminal investment strategy to deceive females if they experience a higher mortality rate than healthy control males [55, 67–71]. For instance, males challenged with nylon implants were found to be more attractive to females than control males, yet they invested less in immune melanization response [69]. Furthermore, males challenged with lipopolysaccharides from the Gram-negative bacteria *Escherichia coli* dissolved in Ringer solution (2.5 mg ml^−1^) were significantly preferred by females over non-challenged control males [71]. Lastly, males challenged with 40 mM of the pro-oxidant Paraquat (PQ) were found to be more attractive to females than control males. However, males exposed to 80 mM of PQ were less attractive than controls [67]. Therefore, *T*. *molitor* presents an intriguing model to explore whether females, akin to males, engage in deceptive behaviour through terminal investment. We predict that females subjected to oxidative stress with PQ or immune challenges with LPS will exhibit greater attractiveness to males and shorter lifespans compared to healthy females. While this outcome is highly probable, the dose of the stressor may determine whether it leads to a terminal investment strategy or honest signalling [67]. In this regard, there are two potential explanations why females may not engage in terminal investment: the dosage could be too low to pose a threat to their survival, or the dosage could be excessively high, surpassing the organism’s capacity to enact it. Hence, we utilized varying levels of LPS or PQ stressors to assess their impact on the likelihood of females exhibiting a terminal investment strategy.

## 2. Material and methods

### (a) Experimental design

*Tenebrio molitor* was reared at 27 ± 0.5°C in complete darkness and provided with *ad libitum* access to a mixture of cornflour and wheat bran (1:3 ratio), supplemented with apple slices every other day [55]. Following this, pupae were individually separated into 12-well boxes (Corning) containing sterile food. Pupae were sexed [72], and adults aged 10–12 days were utilized, as this age aligns with the peak of pheromone production [73]. We selected individuals that were around 13 mm (males: $\bar{x}$ 13.7 ± 0.79 mm; females: $\bar{x}$ 13.6 ± 0.82 mm) and made sure to pair females and males that shared the same size for each of the behavioural trials. This would sometimes reduce the amount of available adults for experiments. Moreover, as some individuals would die before the experiment, the sample size for each treatment slightly varied but we insisted on keeping a minimum of 30 individuals per treatment. We also used individuals who came from the same stock for each treatment to reduce dependent variables.

**Fig 1:**
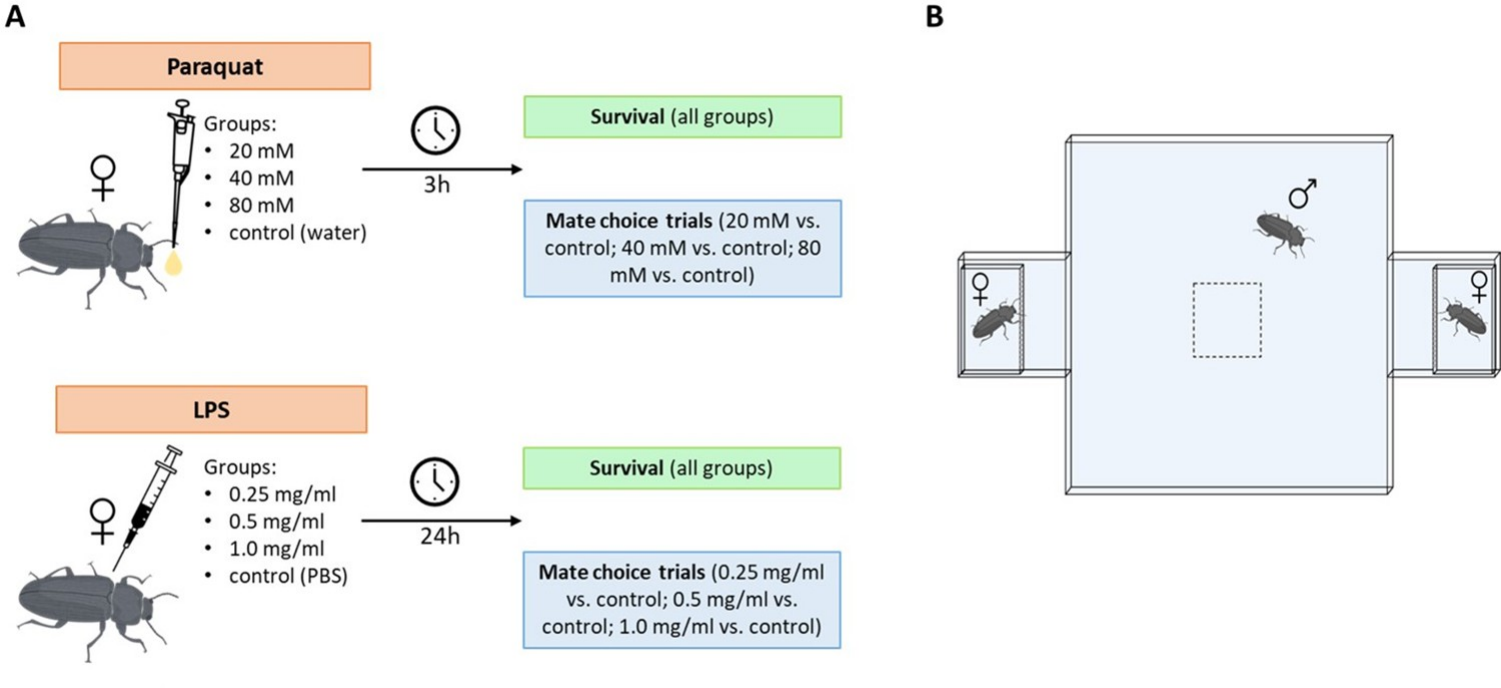
A) Experimental design. Adult females of T. molitor of 12 days old were treated with PQ or LPS and 3 or 24 hours after treatment, respectively, females were tested for survival and/or mate choice. B) Behavioural arena for recording mate choice. The triad is simultaneously placed inside the arena. The males are left for 5 minutes in an enclosure located in the middle of the central box (dotted square in the central part), and then the male is released to walk around the box and enter one of the two chambers where the females are located. The side chambers containing the females have small holes to allow pheromones to pass through and communicate with the male. The insect images were generously provided by Elaine Méndez Muñis.

We injected females with 0.25, 0.5, or 1 mg ml-1 of lipopolysaccharides (LPS) from *Escherichia coli*, diluted in a Ringer solution, as it induces terminal investment in males [69, 74]. Each insect received an injection (using Hamilton syringes of 10 μl) of 1 μl of LPS diluted in Ringer or 1 μl of Ringer alone (control). To induce oxidative stress, we orally administered the herbicide Paraquat (PQ) from Sigma-Aldrich. Each female received 1 μl of PQ diluted in water using a micropipette [55]. We established three experimental groups receiving 20 mM, 40 mM, or 80 mM of PQ diluted in distilled water. The control group received only distilled water. We selected these doses based on preliminary experiments, which demonstrated their effect on chemical communication in *T*. *molitor* [55, 67] and because the same doses induce terminal investment in males [67]. Following treatments, we recorded individual survival every 24 hours (see Fig 1A). Experiments concluded once all individuals in each group had died. Specific sample sizes are provided in the results section.

### (b) Mate choice

To assess female choice, we utilized arenas comprising one large main chamber for the male and two adjacent small chambers for each female [55]. All chambers featured perforated walls to facilitate chemical communication while keeping the females separated from the male (Fig 1B). A habituation period of 5 minutes was allotted before recording mate choice for 10 minutes per trial [55]. Following habituation, males were released to explore, and we documented each visit and its duration for each female he encountered [55]. A visit commenced when the male’s entire body entered the female’s chamber and ceased when his entire body exited [54]. Observers were blinded to each female’s treatment during all trials to maintain data integrity. Fresh individuals were used for each trial, and the chambers were thoroughly cleaned with ethanol between trials.

In all tests, we used triads (Fig 1B) that consisted of one focal male and two females: one control (either injected with Ringer solution or that drank water) and one experimental (injected with 0.25 mg ml-1 or that drank 20 mM, 40 mM or 80mM of PQ). For the PQ treatments, mate choice trials were performed 3 h post-treatment [55], whereas the trials for individuals treated with LPS were performed 24 h post-treatment [71]. Instead of utilizing male pheromone extracts, we chose to expose males to females in order to maintain the continuous production of pheromones or cuticular hydrocarbons (Fig 1B). This method acknowledges potential differences in male and female reactions in the presence of a potential mate, thus offering a more realistic representation of natural scenarios that could result in varying levels of mate attraction [55]. Details regarding sample sizes are provided in the results section. The S1 Video shows an example of mate choice by a male evaluating one female and in the opposite chamber, a rejected male in its chamber.

All animals used in this study were obtained from a culture stock in our laboratory. We ensured to use the minimal number of animals and minimized the harm by using the least invasive methods and choosing oral administration vs injection when possible. For pain management, we placed all individuals in chilled ice to cause a mild sedation.

### (c) Statistical analysis

A Cox survival test was used to compare differences in mortality between treatments. To analyse mate choice, paired t-tests were performed to compare the time females spent visiting each male according to treatment. All tests were carried out in SPSS V.22.0.

## 3. Results

### (a) Survival

#### Immune challenge

Females treated with 0.25 mg ml^-1^ (n = 36) did not differ in survival (X^2^ = 0.36; df = 1; p = 0.54) compared with the control group (n = 35). A similar result was observed between 0.5 mg ml^-1^ (n = 41) versus its control (n = 45) (X^2^ = 0.73; df = 1; p = 0.39) or between 1 mg ml^-1^ (n = 37) versus control (n = 33) (X^2^ = 0.005; df = 1; p = 0.94).

#### Oxidative stress

**Fig 2:**
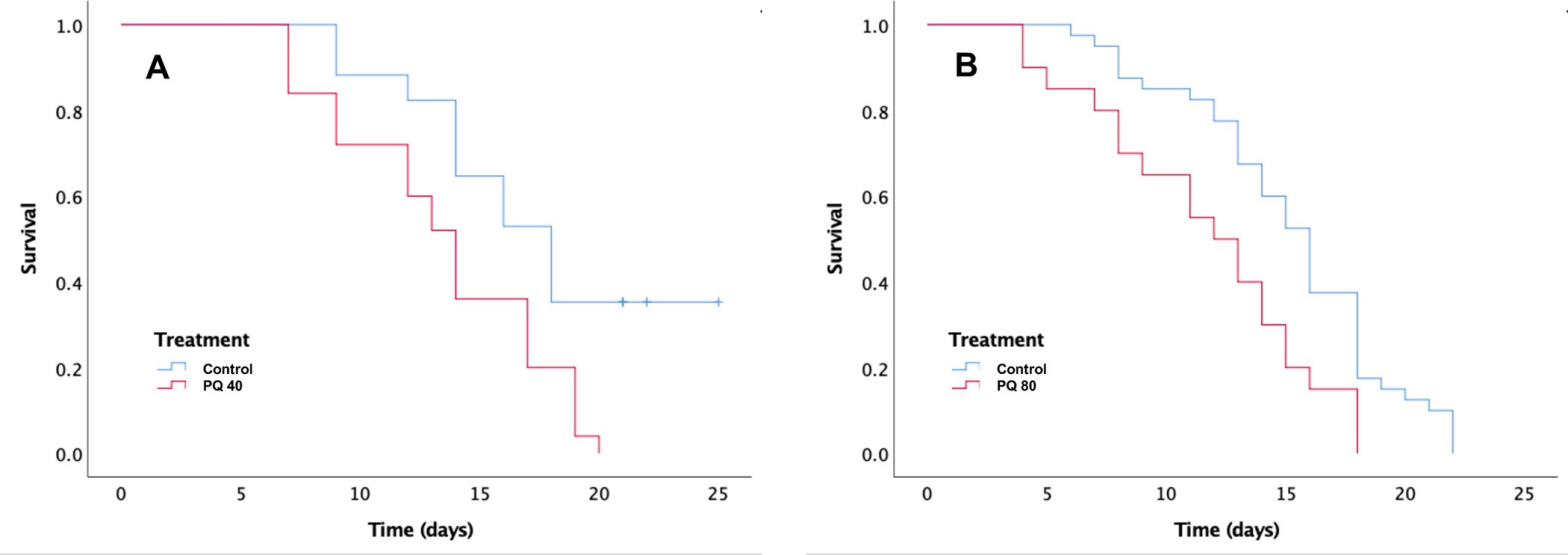
Survival in days of PQ 40 vs Control (A) and PQ 80 vs Control (B). Experiments were conducted independently, with each experimental group compared against its respective control.

Females treated with 20 mM (n = 42) and control females (n = 30) did not differ in survival (X^2^ = 0.08, df = 1; *p =* 0.76). However, females challenged with 40 mM (n = 25) lived less days than their control (n = 17) (X^2^ = 7.2, df = 1; *p =* 0.007) (Fig 2A) and females treated with 80 mM (n = 20) lived less days than their control (n = 40) (X^2^ = 8.5; df = 1; *p =* 0.004) (Fig 2B).

### (b) Mate choice

#### Immune challenge

No significant differences were shown between 0.25 mg ml^-1^ *versus* control (N = 32) (t = -0.15; df = 31; p = 0.88) nor between 0.5 mg ml^-1^ and its control group (N = 33) (t = 0.182; df = 45; p = 0.86) and for 1 mg ml^-1^ and its control (X^2^ = 0.42; df = 1; p = 0.51).

#### Oxidative stress

**Fig 3:**
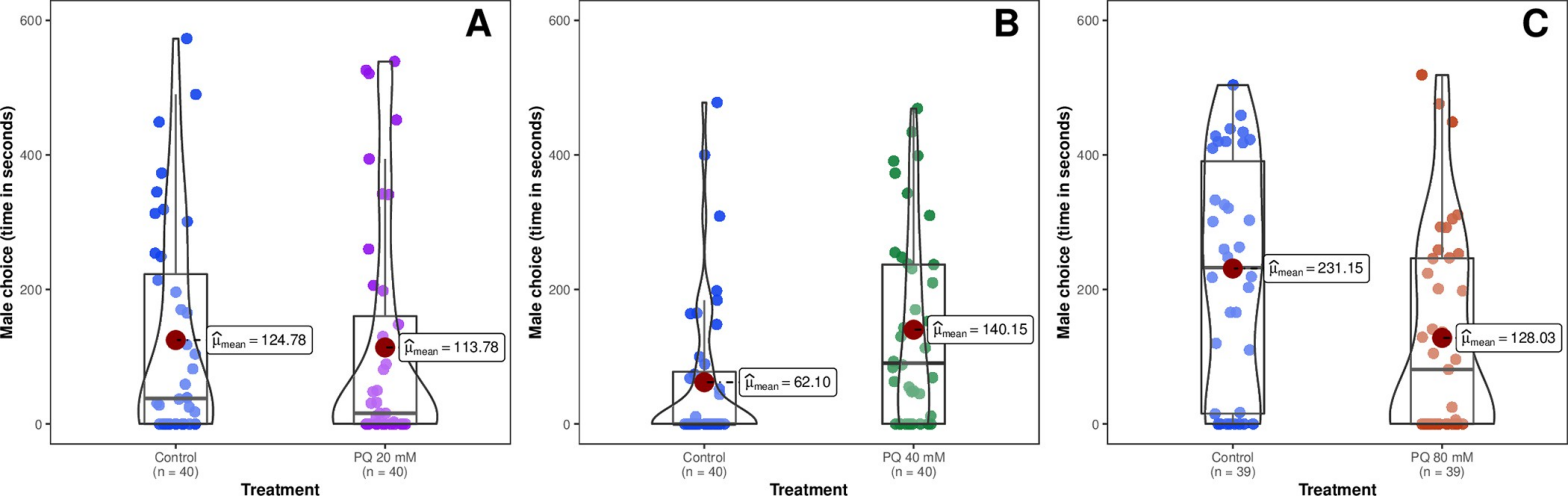
Male choice is measured as the total duration of visits for each treatment. The treatment applied is Paraquat and each graph corresponds to one of the doses administered: A) 40 mM, and B) 80 mM. Bars represent standard errors.

No significant differences were found between 20 mM *versus* control (N = 40) (t = -0.255; df = 39; p = 0.8). However, females treated with 40 mM were preferred over their control (N = 40) (62.1 ± 18.11 s; t = 2.33; df = 39; p = 0.02). Whereas the control females were preferred over those treated with 80 mM (N = 38) (134.17 ± 22.76 s; t = 2.02; df = 37; p = 0.04) (Fig 3).

## 4. Discussion

Since Darwin and Bateman’s proposals [1, 75], research has predominantly focused on males’ strategies to influence females’ decisions. This dominance extends to studies on terminal investment during mate choice, where males are portrayed as deceitful and manipulative to control females’ reproduction [68–71]. Our study reveals that *T*. *molitor* females can employ the terminal investment strategy to deceive males when faced with a 40 mM dose of oxidative stress from PQ. However, when stressors reach a higher level (80 mM PQ), females may struggle to deceive males, prompting them to signal their true poor condition. Females treated with 20 mM of Paraquat survived as well as healthy (control) females, suggesting that this challenge was not sufficiently costly to impair their condition signals. This finding is consistent with our immune challenge results, where the doses females encountered did not compromise their survival, thus they did not use the terminal investment strategy.

Our findings suggest that females exhibit a higher tolerance for oxidative stress and greater resistance to immune challenges compared to males. In a previous study, males treated with 20 mM of Paraquat [55] were less attractive than control males. However, in this study, females’ attractiveness was unaffected by the same dose. This disparity may be explained because females are more resistant than males to oxidative stress [76], potentially accounting for the observed differences in the threshold. Furthermore, numerous studies conducted on male *T*. *molitor* have demonstrated that immune challenges [54–69, 77] elicit a terminal investment response, wherein treated males become more attractive to females than healthy ones. However, the lack of a terminal investment strategy observed in females in this study, despite the administration of LPS at 0.25 mg ml^-1^, is noteworthy. This dose induces changes in cuticular hydrocarbons in males, subsequently increasing their attractiveness to females and eliciting a terminal investment response [71]. The underlying pattern for the relationship between immune response and the presence or absence of sexual hormones presents conflicting findings [78–81]. We propose that males may be less resistant than females to immune challenges and that the presence of Juvenile Hormone (JH) could potentially be a mechanistic link. The JH is present in females and males. However, in females (and not in males) it increases haemocyte number, thereby enhancing their immune response [82]. Consequently, the threshold for triggering terminal investment in females may be higher than in males, which is also partially supported by the lack of survival cost observed in females in this study. However, we do not know the exact mechanisms for this apparent sexual dimorphism in terminal investment and grant further research.

To the best of our knowledge, this is the first study to explore the possibility of females deceiving males to secure a mating partner in the form of terminal investment, as illustrated in Table 1. Engaging in terminal investment could offer females two potential benefits: 1) securing a final mating event before death and/or 2) engaging in reproductive events with one or multiple high-quality males. The latter scenario presents an intriguing phenomenon with significant implications. On one hand, the perceived quality of a mate can drive organisms to allocate greater resources to that reproductive event, as proposed by the differential allocation hypothesis [83]. While our experimental design does not address multiple mating, it is well-documented that females of this species frequently engage in multiple mating to enhance their fecundity [84]. Consequently, it is plausible that females employing the terminal investment strategy may attract more mates due to their heightened attractiveness. In this context, females presenting themselves as high-quality mates could potentially acquire more direct benefits from these mating events. On the other hand, females, males, or both could adjust their investment towards that mating event to optimize the outcome (i.e., offspring quality/quantity) or mitigate the impact of selecting a lower-quality mate. This concept is known as the reproductive compensation hypothesis [83]. This suggests two potential outcomes: 1) if males do not perceive that they are being deceived, their investment in the reproductive event could offset the effects of mating with a lower-quality female; or 2) if the male perceives that he is being deceived, he could strategically modify the quality or quantity of the spermatophore he transfers to the female in order to conserve resources for a future reproductive event. Alternatively, if terminal investment does not affect offspring quality or quantity, cheating males or females may solely benefit from acquiring a mating partner that they would otherwise not obtain if perceived by the opposite sex as being in poor condition. These hypotheses grant further research.

It has been proposed that the terminal investment strategy is dynamic rather than fixed, with a specific temporal window for its occurrence [67, 85, 86]. Our study aligns with findings in males of *T*. *molitor* during mate choice [67], revealing a similarly dynamic strategy in females. This behaviour may be optimal under specific conditions (i.e., at 40 mM PQ), inefficient under high stress (i.e., at 80 mM PQ), or not displayed within a threshold level (i.e. at 20 mM). These findings confirm the importance of considering a gradient in stress levels and simultaneously recording the survival rates of organisms when studying terminal investment in both sexes.

Only a few studies have examined both male and female mate choice within the same species, even though it is estimated to be a widespread phenomenon [9, 87–89]. In insects, male mate choice has been reported in 58 species belonging to 37 families and 11 orders [9], representing only a small fraction of the potential species that may exhibit male mate choice. Mate choice seems to be more widespread than previously thought [16]. Hence, terminal investment in condition-dependent signals by females could be more common than we imagine. It will be important to test whether males, as well as females, can carry out the strategy of terminal investment in such species with mutual mate choice.

Antagonistic sexual conflict theory proposes that females bear elevated costs due to males’ strategies to secure mating partners [11]. However, considering females as the offensive rather than defensive sex also prompts intriguing questions about the costs of deception for males and their potential impacts on subsequent generations. Exploring whether males possess mechanisms to detect deception or memorize partners, akin to females avoiding cheating males (Cordero-Molina et al., Under review), would be insightful. This study underscores that females engage in deceptive behaviours by concealing their condition. Acknowledging that females also possess the ability to deceive their mates for reproductive advantages raises questions about the competition between sexes in reproductive strategies. We should critically examine our sexual and anthropomorphic biases in shaping research questions, as they have influenced studies in sexual selection [90–94] and delve deeper into understanding the differential costs of reproduction and offensive strategies for both sexes. This endeavour has been long overdue since the inception of sexual selection theory [94].

## Supporting information

S1 Video (MP4)

S1 Data Complete data female terminal investment. (XLSX)

## References

[1] Darwin C. The descent of man, and selection in relation to sex. London: Princeton University Press; 1872.

[2] Rosenthal GG, Ryan MJ. Sexual selection and the ascent of women: Mate choice research since Darwin. Science. 2022; 375: eabi6308. doi: 10.1126/science.abi6308 35050648

[3] Holland B, Rice WR. Perspective: chase-away sexual selection: antagonistic seduction versus resistance. Evolution. 1998; 52(1): 1–7. doi: 10.1111/j.1558-5646.1998.tb05132.x 28568154

[4] Chapman T, Arnqvist G, Bangham J, Rowe L. Sexual conflict. Trends Ecol. Evol. 2003; 18(1): 41–47. doi: 10.1016/S0169-5347(02)00004-6

[5] Arnqvist G. Sensory exploitation and sexual conflict. Philos. Trans. R. Soc. Lond., B, Biol. Sci. 2006; 361(1466): 375–386. doi: 10.1098/rstb.2005.1790 16612895 PMC1569614

[6] Andersson MB. Sexual selection. New Jersey: Princeton University Press; 1994.

[7] Krams IA, Mennerat A, Krama T, Krams R, Jõers P, Elferts D, et al. Extra-pair paternity determines cooperation in a bird species. PNAS. 2022; 119(5): e2112004119. doi: 10.1073/pnas.2112004119 35042830 PMC8820227

[8] Fromonteil S, Marie-Orleach L, Winkler L, Janicke T. Sexual selection in females and the evolution of polyandry. PloS Biol. 2023; 21(1): e3001916. doi: 10.1371/journal.pbio.3001916 36626380 PMC9831318

[9] Bonduriansky R. The evolution of male mate choice in insects: a synthesis of ideas and evidence. Biol. Rev. 2001; 76(3): 305–339. doi: 10.1017/s1464793101005693 11569787

[10] Jennions MD, Petrie M. Why do females mate multiply? A review of the genetic benefits. Biol. Rev. 2000; 75(1): 21–64. doi: 10.1017/s0006323199005423 10740892

[11] Arnqvist G, Nilsson T. The evolution of polyandry: multiple mating and female fitness in insects. Anim. Behav. 2000; 60(2): 145–164. doi: 10.1006/anbe.2000.1446 10973716

[12] Zeh JA, Zeh DW. The evolution of polyandry I: intragenomic conflict and genetic incompatibility. Proc. R. Soc. B. 1996; 263(1377): 1711–1717. doi: 10.1098/rspb.1996.0250

[13] Simmons LW. The evolution of polyandry: sperm competition, sperm selection, and offspring viability. Annu. Rev. Ecol. Evol. Syst. 2005; 36: 125–146.

[14] Simmons LW. Sperm Competition and Its Evolutionary Consequences in the Insects. Princeton, New Jersey: Princeton University Press; 2001.

[15] Graves EE, Eadie JM. White eye patches of female wood ducks, Aix sponsa, vary markedly in size and may reflect individual status or condition. Anim. Behav. 2020; 167: 41–53. doi: 10.1016/j.anbehav.2020.06.023

[16] Edward DA, Chapman T. The evolution and significance of male mate choice. Trends Ecol. Evol. 2011; 26(12): 647–654. doi: 10.1016/j.tree.2011.07.012 21890230

[17] Gonzalez-Karlsson A, Golov Y, Steinitz H, Moncaz A, Halon E, Horowitz AR, et al. Males perceive honest information from female released sex pheromone in a moth. Behav. Ecol. 2021; 32(6): 1127–1137. doi: 10.1093/beheco/arab073

[18] Clutton-Brock TH. Reproductive effort and terminal investment in iteroparous animals. Am. Nat. 1984; 123(2): 212–229.

[19] Minchella DJ, Loverde PT. A cost of increased early reproductive effort in the snail Biomphalaria glabrata. Am. Nat. 1981; 118(6): 876–881.

[20] Pugesek BH. Increased reproductive effort with age in the California gull (Larus californicus). Science. 1982; 212(4496): 822–823. doi: 10.1126/science.212.4496.82 17752279

[21] Festa-Bianchet M. Nursing behaviour of bighorn sheep: correlates of ewe age, parasitism, lamb age, birthdate and sex. Anim. Behav. 1988; 36(5): 1445–1454. doi: 10.1016/S0003-3472(88)80215-X

[22] Part T, Gustafsson L, Moreno J. " Terminal investment" and a sexual conflict in the collared flycatcher (Ficedula albicollis). Am.Nat.1992; 140(5): 868–882. doi: 10.1086/285445 19426046

[23] Adamo SA. Evidence for adaptive changes in egg laying in crickets exposed to bacteria and parasites. Anim. Behav. 1999; 57(1): 117–124. doi: 10.1006/anbe.1998.0999 10053078

[24] Sanz JJ, Arriero E, Moreno J, Merino S. Interactions between hemoparasite status and female age in the primary reproductive output of pied flycatchers. Oecologia. 2001; 126: 339–344. doi: 10.1007/s004420000530 28547446

[25] Côté SD, Festa-Bianchet M. Reproductive success in female mountain goats: the influence of age and social rank. Anim. Behav. 2001; 62(1): 173–181. doi: 10.1006/anbe.2001.1719

[26] Ericsson G, Wallin K, Ball JP, Broberg M. Age‐related reproductive effort and senescence in free‐ranging moose, Alces alces. Ecology. 2001; 82(6): 1613–1620. doi: 10.1890/0012-9658(2001)082[1613:ARREAS]2.0.CO;2

[27] Bonneaud C, Mazuc J, Chastel O, Westerdahl H, Sorci G. Terminal investment induced by immune challenge and fitness traits associated with major histocompatibility complex in the house sparrow. Evolution. 2004; 58(12), 2823–2830. doi: 10.1111/j.0014-3820.2004.tb01633.x 15696759

[28] Chadwick W, Little TJ. A parasite-mediated life-history shift in Daphnia magna. Proc. R. Soc. B. 2005; 272(1562): 505–509. doi: 10.1098/rspb.2004.2959 15799946 PMC1578704

[29] Shoemaker KL, Parsons NM, Adamo SA. Mating enhances parasite resistance in the cricket Gryllus texensis. Anim. Behav. 2006; 71(2): 371–380. doi: 10.1016/j.anbehav.2005.05.007

[30] Hanssen SA. Costs of an immune challenge and terminal investment in a long‐lived bird. Ecology. 2006; 87(10): 2440–2446. doi: 10.1890/0012-9658(2006)87[2440:coaica]2.0.co;2 17089653

[31] Blair L, Webster JP. Dose‐dependent schistosome‐induced mortality and morbidity risk elevates host reproductive effort. J. Evol. Biol. 2007; 20(1): 54–61. doi: 10.1111/j.1420-9101.2006.01230.x 17209999

[32] Paitz RT, Harms HK, Bowden RM, Janzen FJ. Experience pays: offspring survival increases with female age. Biol. Lett. 2007; 3(1): 44–46. doi: 10.1098/rsbl.2006.0573 17443962 PMC2373821

[33] Descamps S, Boutin S, Berteaux D, Gaillard JM. Female red squirrels fit Williams’ hypothesis of increasing reproductive effort with increasing age. J. Anim. Ecol. 2007; 76: 1192–1201. doi: 10.1111/j.1365-2656.2007.01301.x 17922715

[34] Marzal A, Bensch S, Reviriego M, Balbontin J, De Lope F. Effects of malaria double infection in birds: one plus one is not two. J. Evol. Biol. 2008; 21(4): 979–987. doi: 10.1111/j.1420-9101.2008.01545.x 18462316

[35] Schwanz LE, Janzen FJ. Climate change and temperature-dependent sex determination: can individual plasticity in nesting phenology prevent extreme sex ratios?. Physiol. Biochem. Zool. 2008; 81(6): 826–834. doi: 10.1086/590220 18831689

[36] Altincicek B, Gross J, Vilcinskas A. Wounding‐mediated gene expression and accelerated viviparous reproduction of the pea aphid Acyrthosiphon pisum. Insect Mol. Biol. 2008; 17(6): 711–716. doi: 10.1111/j.1365-2583.2008.00835.x 18823444

[37] Schwanz LE. Chronic parasitic infection alters reproductive output in deer mice. Behav. Ecol. Sociobiol. 2008; 62: 1351–1358. doi: 10.1007/s00265-008-0563-y

[38] Bercovitch FB, Loomis CP, Rieches RG. Age-specific changes in reproductive effort and terminal investment in female Nile lechwe. J. Mammal. 2009; 90(1): 40–46. doi: 10.1644/08-MAMM-A-124.1

[39] Creighton JC, Heflin ND, Belk MC. Cost of reproduction, resource quality, and terminal investment in a burying beetle. Am. Nat. 2009; 174(5): 673–684. doi: 10.1086/605963 19775240

[40] Clutton-Brock TH, Guinness FE, Albon SD. Red deer: behavior and ecology of two sexes. Chicago: University of Chicago press; 1982.

[41] Barrett EL, Hunt J, Moore AJ, Moore PJ. Separate and combined effects of nutrition during juvenile and sexual development on female life-history trajectories: the thrifty phenotype in a cockroach. Proc. R. Soc. B. 2009; 276(1671): 3257–3264. doi: 10.1098/rspb.2009.0725 19553255 PMC2817170

[42] Cotter SC, Simpson SJ, Raubenheimer D, Wilson K. Macronutrient balance mediates trade-offs amongst competing life-history and immune traits. Funct. Ecol. 2011; 25(1): 186–198.

[43] Hoffman CL, Higham JP, Mas-Rivera A, Ayala JE, Maestripieri D. Terminal investment and senescence in rhesus macaques (Macaca mulatta) on Cayo Santiago. Behav. Ecol. 2010; 21(5): 972–978. doi: 10.1093/beheco/arq098 22475990 PMC2920293

[44] Bowers EK, Smith RA, Hodges CJ, Zimmerman L M, Thompson CF, Sakaluk SK. Sex-biased terminal investment in offspring induced by maternal immune challenge in the house wren (Troglodytes aedon). Proc. R. Soc. B. 2012; 279(1739): 2891–2898. doi: 10.1098/rspb.2012.0443 22456887 PMC3367793

[45] Heinze J, Schrempf A. Terminal investment: individual reproduction of ant queens increases with age. PLoS ONE. 2012; 7(4): e35201. doi: 10.1371/journal.pone.0035201 22509399 PMC3324418

[46] Bowers EK, Bowden RM, Sakaluk SK, Thompson CF. Immune activation generates corticosterone-mediated terminal reproductive investment in a wild bird. Am. Nat. 2015; 185(6): 769–783. doi: 10.1086/681017 25996862 PMC4443487

[47] Staudacher H. Menken SB Groot AT. Effects of immune challenge on the oviposition strategy of a noctuid moth. J. Evol. Biol. 2015; 28(8): 1568–1577. doi: 10.1111/jeb.12677 26086071

[48] Brannelly LA, Webb R, Skerratt LF, Berger L. Amphibians with infectious disease increase their reproductive effort: evidence for the terminal investment hypothesis. Open Biol. 2016; 6(6):150251. doi: 10.1098/rsob.150251 27358291 PMC4929933

[49] Giehr J, Grasse AV, Cremer S, Heinze J, Schrempf A. Ant queens increase their reproductive efforts after pathogen infection. R. Soc. Open Sci. 2017; 4(7): 170547. doi: 10.1098/rsos.170547 28791176 PMC5541571

[50] Guivier E, Lippens C, Faivre B, Sorci G. Plastic and micro-evolutionary responses of a nematode to the host immune environment. Exp. Parasitol. 2017; 181: 14–22. doi: 10.1016/j.exppara.2017.07.002 28733132

[51] Adamo SA, McKee R. Differential effects of predator cues versus activation of fight-or-flight behaviour on reproduction in the cricket Gryllus texensis. Anim. Behav. 2017; 134: 1–8. doi: 10.1016/j.anbehav.2017.09.027

[52] Sköld-Chiriac S, Nilsson JÅ, Hasselquist D. Immune challenge induces terminal investment at an early breeding stage in female zebra finches. Behav. Ecol. 2019; 30(1): 166–171. doi: 10.1093/beheco/ary147

[53] Hudson AL, Moatt JP, Vale PF. Terminal investment strategies following infection are dependent on diet. J. Evol. Biol. 2020; 33(3): 309–317. doi: 10.1111/jeb.13566 31705829

[54] Contreras-Garduño J, Méndez-López TT, Patiño-Morales A, González-Hernández GA, Torres-Guzmán JC, Krams I, Ruiz-Guzmán G. The costs of the immune memory within generations. Sci. Nat. 2019; 106: 1–9. doi: 10.1007/s00114-019-1657-2 31758265

[55] Ruiz‐Guzmán G, Cordero‐Molina S, Krams I, Contreras‐Garduño J. Interactions between oxidative stress and attractiveness to mates and individual mate choice in the beetle Tenebrio molitor. Ethol. 2021; 127(2): 109–116. doi: 10.1111/eth.13108

[56] Griffith OL, Vakili R, Currie RW, Vanderwel D. The effect of mating on the sex pheromone system of the yellow mealworm beetle, Tenebrio molitor L. (Coleoptera: Tenebrionidae). J. Stored Prod. Res. 2020; 86: 101572. doi: 10.1016/j.jspr.2020.101572

[57] Bryning GP, Chambers J, Wakefield ME. Identification of a sex pheromone from male yellow mealworm beetles, Tenebrio molitor. J. Chem. Ecol. 2005; 31: 2721–2730. doi: 10.1007/s10886-005-7622-x 16273437

[58] Rantala MJ, Jokinen I, Kortet R, Vainikka A, Suhonen J. Do pheromones reveal male immunocompetence?. Proc. R. Soc. B. 2002; 269(1501): 1681–1685. doi: 10.1098/rspb.2002.2056 12204128 PMC1691089

[59] Hurd H, Parry G. Metacestode-induced depression of the production, and responses to, sex pheromone in the intermediate host Tenebrio molitor. J. Invertebr. Pathol. 1991; 58(1): 82–87. doi: 10.1016/0022-2011(91)90165-M 1885925

[60] Worden BD, Parker PG, Pappas PW. Parasites reduce attractiveness and reproductive success in male grain beetles. Anim. Behav. 2000; 59(3), 543–550. doi: 10.1006/anbe.1999.1368 10715176

[61] Rantala MJ, Kortet R, Kotiaho JS, Vainikka A, Suhonen J. Condition dependence of pheromones and immune function in the grain beetle Tenebrio molitor. Funct. Ecol. 2003; 17: 534–540. doi: 10.1046/j.1365-2435.2003.00764.x

[62] Rantala MJ, Vainikka A, Kortet R. The role of juvenile hormone in immune function and pheromone production trade-offs: a test of the immunocompetence handicap principle. Proc. R. Soc. B. 2003; 270(1530): 2257–2261. doi: 10.1098/rspb.2003.2472 14613612 PMC1691508

[63] Worden BD, Parker PG. Females prefer noninfected males as mates in the grain beetle Tenebrio molitor: evidence in pre-and postcopulatory behaviours. Anim. Behav. 2005; 70(5): 1047–1053. doi: 10.1016/j.anbehav.2005.01.023

[64] Tanaka Y, Honda H, Ohsawa K, Yamamoto I. A sex attractant of the yellow mealworm, Tenebrio molitor L., and its role in the mating behavior. J. Pestic. Sci. 1986; 11(1): 49–55. doi: 10.1584/jpestics.11.49

[65] Carazo P, Sanchez E, Font E, Desfilis E. Chemosensory cues allow male Tenebrio molitor beetles to assess the reproductive status of potential mates. Anim. Behav. 2004; 68(1): 123–129. doi: 10.1016/j.anbehav.2003.10.014

[66] Vanderwel D, Bacala CH, Bacala R, Currie RW. Clarification of the role of 4-methylnonanol, female-produced sex pheromone of the yellow mealworm beetle, Tenebrio molitor (Coleoptera: Tenebrionidae). J. Stored Prod. Res. 2017; 70: 60–64. doi: 10.1016/j.jspr.2016.12.001

[67] Cordero-Molina S, Mendoza L, Alvarado Y, Krams I, Contreras-Garduño J. Exploring the males’ terminal investment strategy: Impact of the dose of stress and the time lapse between stress and mating. Ecol. Entomol. 2024; 49(1): 21–30. doi: 10.1111/een.13275

[68] Sadd B, Holman L, Armitage H, Lock F, Marland R, Siva-Jothy MT. Modulation of sexual signalling by immune challenged male mealworm beetles (Tenebrio molitor, L.): evidence for terminal investment and dishonesty. J. Evol. Biol. 2006; 19(2): 321–325. doi: 10.1111/j.1420-9101.2005.01062.x 16599907

[69] Krams I, Daukšte J, Kivleniece I, Krama T, Rantala MJ, Ramey G, et al. Female choice reveals terminal investment in male mealworm beetles, Tenebrio molitor, after a repeated activation of the immune system. J. Insect Sci. 2011; 11(1): 56. doi: 10.1673/031.011.5601 21864151 PMC3281432

[70] Krams IA, Krama T, Moore FR, Rantala MJ, Mänd R, Mierauskas P, et al. Resource availability as a proxy for terminal investment in a beetle. Oecologia. 2015; 178: 339–345. doi: 10.1007/s00442-014-3210-5 25582868

[71] Nielsen ML, Holman L. Terminal investment in multiple sexual signals: immune‐challenged males produce more attractive pheromones. Funct. Ecol. 2012; 26(1): 20–28. doi: 10.1111/j.1365-2435.2011.01914.x

[72] Bhattacharya AK, Ameel JJ, Waldbauer GP. A method for sexing living pupal and adult yellow mealworms. Ann. Entomol. Soc. Am. 1970; 63(6): 1783–1783. doi: 2443/10.1093/aesa/63.6.1783

[73] Happ GM, Wheeler J. Bioassay, preliminary purification, and effect of age, crowding, and mating on the release of sex pheromone by female Tenebrio molitor. Ann. Entomol. Soc. Am. 1969; 62(4): 846–851. doi: 10.1093/aesa/62.4.846

[74] Moret Y. ‘Trans-generational immune priming’: specific enhancement of the antimicrobial immune response in the mealworm beetle, Tenebrio molitor. Proc. R. Soc. B. 2006; 273(1592): 1399–1405. doi: 10.1098/rspb.2006.3465 16777729 PMC1560290

[75] Bateman AJ. Intra-sexual selection in Drosophila. Heredity. 1948; 2(3): 349–368. doi: 10.1038/hdy.1948.21 18103134

[76] Niveditha S, Deepashree S, Ramesh SR, Shivanandappa T. Sex differences in oxidative stress resistance in relation to longevity in Drosophila melanogaster. J. Comp. Physiol. B. 2017; 187: 899–909. doi: 10.1007/s00360-017-1061-1 28261744

[77] Kivleniece I, Krams I, Daukšte J, Krama T, Rantala MJ. Sexual attractiveness of immune-challenged male mealworm beetles suggests terminal investment in reproduction. Anim. Behav. 2010; 80(6): 1015–1021. doi: 10.1016/j.anbehav.2010.09.004

[78] Folstad I, Karter AJ. Parasites, bright males, and the immunocompetence handicap. Am. Nat. 1992; 139(3): 603–622. doi: 10.1086/285346

[79] Zuk M, Simmons LW, Rotenberry JT, Stoehr AM. Sex differences in immunity in two species of field crickets. Can. J. Zool. 2004; 82(4): 627–634. doi: 10.1139/z04-032

[80] Nunn CL, Lindenfors P, Pursall ER, Rolff J. On sexual dimorphism in immune function. Philos. Trans. R. Soc. B. 2009; 364(1513): 61–69. doi: 10.1098/rstb.2008.0148 18926977 PMC2666693

[81] Rantala MJ, Dubovskiy IM, Pölkki M, Krama T, Contreras-Garduño J, Krams IA. Effect of juvenile hormone on resistance against entomopathogenic fungus Metarhizium robertsii differs between sexes. J. Fungi. 2020; 6(4): 298. doi: 10.3390/jof6040298 33227937 PMC7711818

[82] Amaro-Sánchez T, Ruiz-Guzmán G, Hernández-Martínez S, Krams I, Rantala MJ, Contreras-Garduño J. Effect of juvenile hormone on phenoloxidase and hemocyte number: The role of age, sex, and immune challenge. Comp. Biochem. Physiol. B, Biochem. Mol. Biol. 2023; 265: 110827. doi: 10.1016/j.cbpb.2023.110827 36610635

[83] Burley N. The differential-allocation hypothesis: an experimental test. Am. Nat. 1988; 132(5): 611–628. doi: 10.1086/284877

[84] Drnevich JM, Papke RS, Rauser CL, Rutowski RL. Material benefits from multiple mating in female mealworm beetles (Tenebrio molitor L.). J. Insect Behav. 2001; 14: 215–230. doi: 10.1023/A:1007889712054

[85] Duffield KR, Bowers EK, Sakaluk SK, Sadd, BM. A dynamic threshold model for terminal investment. Behav. Ecol. Sociobiol. 2017; 71: 1–17. doi: 10.1007/s00265-017-2416-z 30002566 PMC6039117

[86] Foo YZ, Lagisz M, O’Dea RE, Nakagawa S. The influence of immune challenges on the mean and variance in reproductive investment: a meta-analysis of the terminal investment hypothesis. BMC Biol. 2023; 21(1): 107. doi: 10.1186/s12915-023-01603-4 37173684 PMC10176797

[87] Pitafi KD, Simpson R, Day TH. Male mate choice for fecund females in seaweeds flies. Pak. J. Zool. 1995; 27: 233–240.

[88] Johnstone RA, Reynolds JD, Deutsch JC. Mutual mate choice and sex differences in choosiness. Evolution. 1996; 50(4): 1382–1391. doi: 10.1111/j.1558-5646.1996.tb03912.x 28565695

[89] Kokko H, Johnstone RA. Why is mutual mate choice not the norm? Operational sex ratios, sex roles and the evolution of sexually dimorphic and monomorphic signalling. Philos. Trans. R. Soc. B. 2002; 357(1419): 319–330. doi: 10.1098/rstb.2001.0926 11958700 PMC1692955

[90] Birkhead TR, Pizzari T. Postcopulatory sexual selection. Nat. Rev. Genet. 2002; 3(4): 262–273. doi: 10.1038/nrg774 11967551

[91] Snyder BF, Gowaty PA. A reappraisal of Bateman’s classic study of intrasexual selection. Evolution. 2007; 61(11): 2457–2468. doi: 10.1111/j.1558-5646.2007.00212.x 17725639

[92] Clutton-Brock T. Sexual selection in males and females. Science. 2007; 318(5858): 1882–1885. doi: 10.1126/science.1133311 18096798

[93] Roughgarden J, Akçay E. Do we need a Sexual Selection 2.0?. Anim. Behav. 2010; 79(3): e1–e4. doi: 10.1016/j.anbehav.2009.06.006

[94] Ah-King M. The history of sexual selection research provides insights as to why females are still understudied. Nat. Commun. 2022; 13(1): 6976. doi: 10.1038/s41467-022-34770-z 36379954 PMC9666445

